# ZnO Tetrapods for Label-Free Optical Biosensing: Physicochemical Characterization and Functionalization Strategies

**DOI:** 10.3390/ijms24054449

**Published:** 2023-02-23

**Authors:** Monica Terracciano, Simas Račkauskas, Andrea Patrizia Falanga, Sara Martino, Giovanna Chianese, Francesca Greco, Gennaro Piccialli, Guido Viscardi, Luca De Stefano, Giorgia Oliviero, Nicola Borbone, Ilaria Rea

**Affiliations:** 1 Department of Pharmacy, University of Naples Federico II, Via Domenico Montesano 49, 80131 Naples, Italy; 2 Institute of Materials Science, Kaunas University of Technology, 51423 Kaunas, Lithuania; 3 Unit of Naples, National Research Council, Institute of Applied Sciences and Intelligent Systems, Via Pietro Castellino 111, 80131 Naples, Italy; 4 Department of Precision Medicine, University of Campania “Luigi Vanvitelli”, 80138 Naples, Italy; 5 Department of Chemistry, NIS Interdepartmental Centre, University of Turin, Via Pietro Giuria 7, 10125 Turin, Italy; 6 Department of Molecular Medicine and Medical Biotechnologies, University of Naples Federico II, Via S. Pansini 5, 80131 Naples, Italy

**Keywords:** surface characterization, quantum yield, label-free detection, zinc oxide nanostructure, surface functionalization

## Abstract

In this study, we fabricated three different ZnO tetrapodal nanostructures (ZnO-Ts) by a combustion process and studied their physicochemical properties by different techniques to evaluate their potentiality for label-free biosensing purposes. Then, we explored the chemical reactivity of ZnO-Ts by quantifying the available functional hydroxyl groups (–OH) on the transducer surface necessary for biosensor development. The best ZnO-T sample was chemically modified and bioconjugated with biotin as a model bioprobe by a multi-step procedure based on silanization and carbodiimide chemistry. The results demonstrated that the ZnO-Ts could be easily and efficiently biomodified, and sensing experiments based on the streptavidin target detection confirmed these structures’ suitability for biosensing applications.

## 1. Introduction

The increasing interest in the earliest diagnosis has led to a considerable expansion of the biosensing field, with the continuous search for the most suitable materials for developing high-performance and low-cost detection devices [1,2]. Bulk materials lack important physicochemical properties (e.g., high surface-to-volume ratio, chemical reactivity, optical and electrical properties) needed for the development of biosensors, thus promoting the synthesis of nanostructured materials. Due to their distinct physicochemical properties, nanomaterials are appropriate as building blocks for biosensor development, providing high sensitivity and a valuable platform for analyzing single-molecular activity [3]. Among different materials, zinc oxide (ZnO) has arisen as a versatile, low-cost, and abundant metal oxide semiconductor useful to synthesize new transducer platforms for biosensor development [4,5,6].

Various forms of ZnO nanostructures, such as nanowires, nanotubes, tetrapods (Ts), nanorods, etc., are obtained by simple synthetic processes from low-cost materials [7,8]. The easy tuning of nanostructures’ morphology can significantly alter their physicochemical properties, such as enhancing their surface area and promoting variations in their optical, electrical, and electrochemical responses [9]. In addition, their properties, such as their ultraviolet (UV) light sensitivity, wide bandgap (~3.37 eV), high electron transfer capability (~60 meV), and high isoelectric point (IEP ~ 9.5), make them promising materials for biosensing applications [10]. Thanks to the great progress in nanotechnology and material science, new technological methods have been developed to fabricate nanostructured ZnO templates with high surface area and advanced properties. In a previous work, we demonstrated that the emerging ZnO tetrapodal structures (ZnO-Ts), compared to ZnO nanorods and nanoparticles, have better electrochemical properties as a transducer platform for biosensing applications [10]. ZnO-Ts are characterized by four connective arms protruding from the core center at an average angle of ~110°. Their characteristic three-dimensional structure makes them better than one-dimensional nanostructures for biosensing applications due to the requisite high conductivity, limited agglomeration, and easy fabrication of electrodes and sensing devices, opening the way for the development of a label-free, multiparametric-read-out platform [4,5].

A nanostructured matrix used in biosensor development acts as both a solid support, which is used for the platform for the immobilization of sensing biomolecules (i.e., bioprobes) and as a transducer, which is able to convert target detection into an analytical read-out signal [11]. The bioprobe label can be ascribed to a variety of biomolecules spanning among simple or complex structures: single-stranded (DNA and RNA) oligonucleotides, aptamers, peptide nucleic acids (PNAs), proteins, enzymes, antibodies, peptides, etc. [7,12,13,14,15,16,17].

The fabrication of a new generation of hybrid biosensors in which biological or bio-inspired molecules are fully integrated with transducer platforms strongly depends on the functionalization and bioconjugation strategies of the device’s surface [11,18]. Therefore, not only are the support’s physical and chemical properties fundamental for determining the bioprobe immobilization method but the stability of the transducer surface during the functionalization and detection procedures must also be considered [19].

In this context, we explored ZnO-Ts as potential nanostructures for the development of label-free optical biosensors, paying particular attention to their physicochemical properties.

Three different ZnO-T samples (SH0, SH1, and SH2), synthesized by the combustion process and separated by centrifugation method in different size fractions, were characterized by dynamic light scattering (DLS), transmission electron microscopy (TEM), Brunauer-Emmett-Teller (BET) analyses, Fourier-transform infrared spectroscopy (FTIR), UV-vis spectroscopy, spectrofluorimetry, steady-state photoluminescence (PL), and fluorescence microscopy.

The use of ZnO-Ts as transducers in biosensor development requires the creation of coupling points for the biomolecules’ immobilization (the so-called bioconjugation process), preserving the specific functionalities of biological receptors through good control of their orientation and organization on the inorganic surface [20]. To this aim, to estimate the number of hydroxyl groups (–OH) exposed on the tetrapods’ surface potentially available for functionalization with the bioprobe, we functionalized the nanostructures’ surface with 5′-DMT-3′-phosphoramidite-thymidine nucleotides via phosphoramidite chemistry and quantified, by colorimetric analysis, the amount of the DMT^+^ cation released in solution after coupling and acid treatment, which corresponds to the amount of reactive –OH groups exposed on the Ts’ surface [21]. Then, the best T sample in terms of colloidal stability, PL quantum yield, and chemical reactivity was chemically modified and bioconjugated with biotin (used here as the bioprobe model), experiencing chemical protocols able to preserve the physicochemical properties of the matrix. To this end, ZnO nanostructures were chemically modified with a multi-step procedure consisting of a silanization step by a 3-(aminopropyl)triethoxysilane compound (APT) followed by biotin conjugation by carbodiimide chemistry [22,23,24]. The efficacy of the resulting ZnO-biotin biosensor was evaluated by exploiting the real-time detection of streptavidin using optical methods based on steady-state PL and fluorescence microscopy.

## 2. Results and Discussion

### 2.1. Physicochemical Characterization of Bare ZnO-Ts

**Figure 1:**
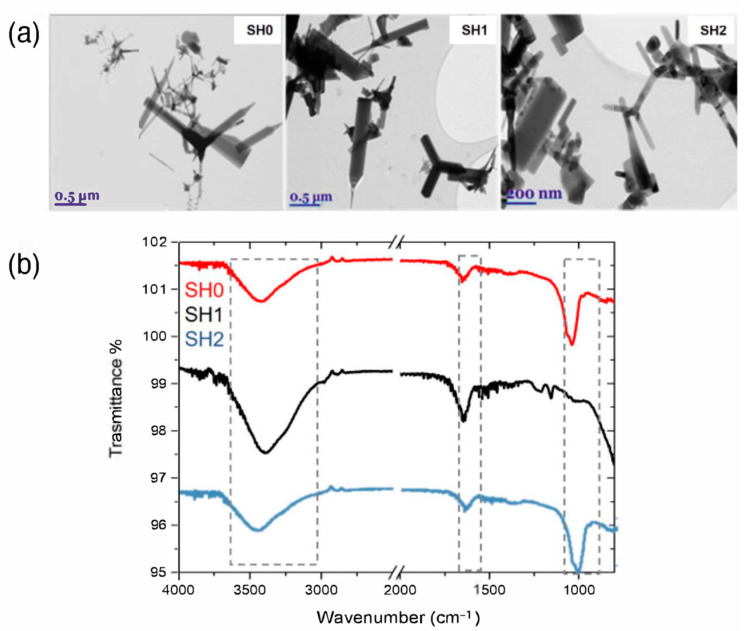
Morphological and chemical characterization of SH0, SH1, and SH2 ZnO-T samples. (**a**) TEM images showing the characteristic tetrahedral structure of ZnO-Ts. (**b**) ATR-FTIR spectra.

ZnO-Ts were obtained by a combustion method and then separated by size into three fractions, SH0, SH1, and SH2, as described in Section 3.2. Centrifugation separates ZnO-Ts into fractions by their mass; therefore, the largest ZnO-T structures are obtained in fraction SH1, the smallest are obtained in fraction SH2, and the initial fraction, SH0, has both large SH1 and small SH2 fractions. The morphology of as-synthetized ZnO-Ts was investigated by transmission electron microscopy (TEM). The analysis revealed nanostructures characterized by the typical tetrapodal shape consisting of four connected nanorods (legs) with a diameter of 20–100 nm and a length of 100–1000 nm (Figure 1a). Different tetrapods’ leg morphology could be connected to local fluctuations of synthesis conditions in the combustion chamber. The sample SH2 showed the smallest diameters and lengths; SH1 showed the biggest diameters and lengths, while SH0 had both small and big ZnO-Ts. A BET analysis (Figure S1) revealed the increase in the surface area along the series SH1 < SH0 < SH2, with corresponding values of 7.1 ± 0.1, 8.9 ± 0.1, and 10.8 ± 0.2 m^2^ g^−1^.

The surface chemistry of bare ZnO-T samples was investigated by attenuated total reflectance Fourier-transform infrared (ATR–FTIR) spectroscopy. The spectra reported in Figure 1b show absorption bands at 3500 cm^−1^ and 1050 cm^−1^ due to the O–H stretching and bending vibrations, respectively. The band at 1630 cm^−1^, attributed to zinc carboxylate due to the synthesis process, is also evident [25,26].

Dynamic light scattering (DLS) analysis was performed to evaluate the stability of colloidal suspensions by measuring the hydrodynamic diameter (size), polydispersity index (PdI), and surface charge (zeta potential) of the three ZnO-T bare samples. To this aim, ZnO-Ts were dispersed in ultra-pure water (pH 7) at a concentration of 0.05 mg mL^−1^ following the procedure described in Section 3.3. SH0 showed a smaller size, lower PdI, and higher zeta potential than the other samples, thus confirming the good homogeneity and stability of the SH0 ZnO-T colloidal suspension (Table 1). However, we observed that the bare ZnO-Ts had a very high tendency to agglomerate in deionized water, leading to a larger size in the DLS analyses compared to the real size determined by TEM due to the lack of stabilizing surface chemistry [27]. Therefore, it was necessary to chemically stabilize the ZnO-Ts surface for biosensing purposes.

**Figure 2:**
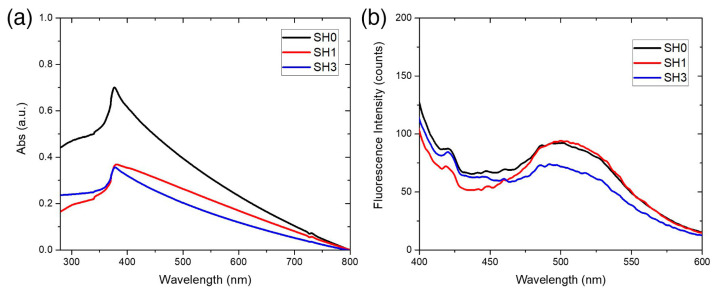
Optical characterization of ZnO-T samples SH0, SH1, and SH2. (**a**) UV-Vis absorption spectra showing an absorption peak at 370 nm. (**b**) Fluorescence spectra of the samples excited at 370 nm.

The absorbance of ZnO-Ts dispersed in PBS-T was investigated by UV-vis spectroscopy in the wavelength range included between 280 and 800 nm (Figure 2a). In this interval, an absorption peak at 370 nm was observed; the peak can be ascribed to the intrinsic bandgap absorption of ZnO due to the electron transitions from the valence band to the conduction band (O2p → Zn3d) [28]. The fluorescence emission of ZnO-Ts in PBS-T was studied under an excitation light of 370 nm. The emission spectra of samples SH0, SH1, and SH2, reported in Figure 2b, are characterized by a broad band centered at 500 nm due to the defects present in the materials, in particular oxygen vacancies and –OH groups [29,30]. A weak peak at about 420 nm can also be observed. This peak can be attributed to the transitions from Zn interstitials to the valence band [31].

**Figure 3:**
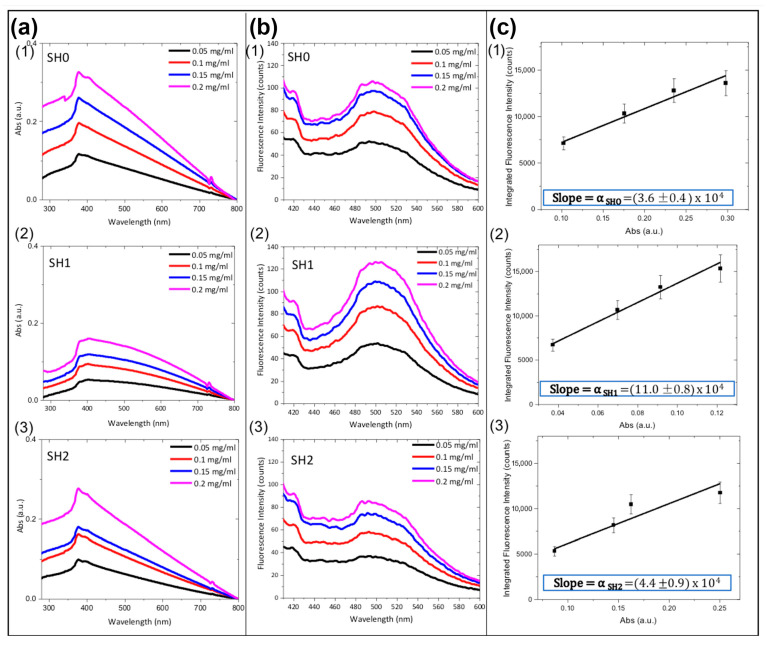
(**a**) Absorption and (**b**) fluorescence spectra (λ_exc_ = 370 nm) of ZnO-Ts (SH0, SH1, SH2) at different concentrations in PBS-T. (**c**) Corresponding integrated fluorescence intensity versus absorbance at 370 nm; the slopes of the curves were used for calculating the quantum yield (QY) of each sample.

After this preliminary optical characterization, the fluorescence quantum yields (QYs) of SH0, SH1, and SH2 were determined by measuring the absorbance and the fluorescence intensity (λ_exc_ = 370 nm) of the samples dispersed in PBS-T at different concentrations ranging from 0.05 to 0.2 mg mL^−1^ (Figure 3a,b). QYs were estimated relative to Hoechst 33342 (Hoechst) used as a standard dye (the QY of Hoechst in DMF is 35% and calculated using the following equation:

(1) $${\left.\frac{{\mathrm{QY}}_{\mathrm{ZnO}\;\mathrm{Tds}}}{n_{\mathrm{PBS}-T}^{2}\alpha_{\mathrm{ZnO}\;\mathrm{Tds}}}\right|}_{\lambda_{\mathrm{exc}}}={\left.\frac{{\mathrm{QY}}_{\mathrm{Hoechst}}}{n_{\mathrm{DMF}}^{2}\alpha_{\mathrm{Hoechst}}}\right|}_{\lambda_{\mathrm{exc}}}$$

where n is the refractive indices of the media (PBS-T or DMF) and α represents the ratio between the integrated fluorescence intensity and the absorbance at λ = 370 nm) [32,33]. The coefficients αSH0 = (3.6 ± 0.4) × 10^4^, αSH1 = (11.0 ± 0.8) × 10^4^, and αSH2 = (4.4 ± 0.9) × 10^4^ were obtained via linear regression from the plots of integrated fluorescence intensity versus absorbance for SH0, SH1, and SH2, respectively (Figure 3c). The coefficient α_Hoechst_ = (2.3 ± 0.1) × 10^5^ was calculated for Hoechst in DMF. Using n_PBS−T_ = 1.33 and n_DMF_ = 1.43 as refractive indices. The QY values reported in Table 2 were determined from Equation (1).

### 2.2. Nucleotides Conjugation to ZnO-Ts by Phosphonamidite Chemistry and Quantification of Reactive Hydroxyl Groups on ZnO-T Surface

**Figure 4:**
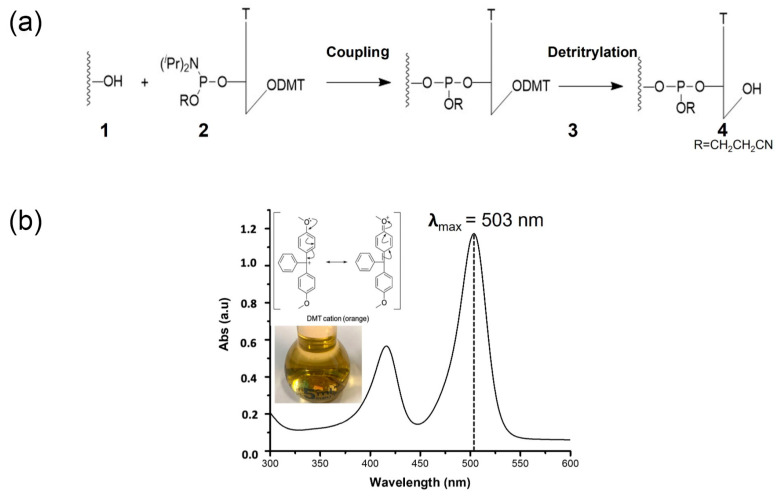
(**a**) Functionalization scheme of ZnO-Ts (**1**) with 5′-DMT-3′-phosphoramidite-thymidine nucleotide (**2**) via coupling reaction; the removal of 5′-DMT from thymidine nucleotide-bounded on ZnO-Ts surface (**3**) via detritylation reaction provided the ZnO-Ts surface (**4**). (**b**) Representative images of DMT cation bright red-orange colored solution resulting from detritylation where the quantity of the released DMT cation is measured by UV-vis spectroscopy at 503 nm.

Proper surface chemical functionalization of the transducer material is paramount for biosensor development. The transducer surface is frequently functionalized to improve its physicochemical properties and enrich its functionalities. Therefore, the precise quantification of available functional groups on the transducer surface is fundamental to adequately control the chemical modification [22]. We determined the number of reactive –OH groups exposed on the bare ZnO-Ts’ surface using a non-conventional methodology based on nucleotide derivatization followed by a colorimetric assay. To this aim, ZnO-Ts (1, Figure 4a) were left to react with tetrazole-activated 5′-DMT-3′-phosphoramidite-thymidine nucleotides (2, Figure 4a) using the well-known phosphoramidite chemistry. This reaction allowed the binding of T nucleotides on the ZnO surface by the fast formation of 3′-phosphite-triester groups with the –OH groups exposed on the ZnO surface (3, Figure 4a). The amount of the bonded nucleotides, which reflects the amount of the reactive –OH groups on the ZnO-Ts’ surface, was assessed by spectrophotometrically quantifying the amount of the 5′-dimethoxytrityl cations (DMT^+^) released from the ZnO-T-bound 5′-DMT-protected nucleotides (3, Figure 4a) using a solution of dichloroacetic acid in dichloromethane (3% *w/w*). The release of the DMT^+^ cations generates a bright red-orange colored solution (A_max_ = 503 nm) whose color intensity is directly proportional to the content of –OH groups and quantifiable by the Lambert–Beer law (ε = 71,700 M^−1^ cm^−1^) as shown in Figure 4b [34]. The amount of DMT^+^ cation released in solution was 3.9 ± 0.2, 3.5 ± 0.6, and 1.8 ± 0.5 µmol mg^−1,^ respectively, for SH0, SH1, and SH2 (Table 3).

### 2.3. Biofunctionalization of ZnO-Ts Surface and Sensing Experiment

The selectivity of the device is another key issue in biosensor development. Selectivity is achievable by using specific bioprobes that can be either directly grown into the PSi matrix (in situ synthesis) or synthetized ex situ and then immobilized on the surface via electrostatic or covalent interactions [12,13,15]. This delicate step might affect the mobility, conformation, and functionalities of the selected bioprobes; therefore, various chemical strategies were developed to preserve the bioprobe’s functionality for biosensing applications [2].

Another important aspect to consider in biosensor development is the pH of aqueous-based solutions used during biofunctionalization procedures. The pH could hugely impact the ZnO-based nanostructures and their properties considering that ZnO is an amphoteric oxide easily dissolvable in both acid and basic conditions. The metal oxide ZnO in water solution undergoes hydrolysis, creating a hydroxide coating on its surface (≡M–OH) [19]. In acid conditions, the H_3_O^+^ ions react with the ZnO surface, causing the dissolution of nanostructures with rapid release of Zn^2+^(aq) in the alkaline condition (pH higher than 8.5) also occurs due to the dissolution of ZnO nanostructures related to their hydroxide which produces soluble species in the form of hydroxyl complexes such as Zn(OH)_2_(aq). The mechanisms of ZnO-based structures’ dissolution in acid and alkaline conditions are described in detail in the “Supporting Information”. Considering the results reported in the literature, the best working condition to develop a ZnO-based biosensor preserving the physicochemical properties is to use mild alkaline pH solutions [19,35,36].

The SH0 sample was chosen for the experiments of bioconjugation and optical sensing due to two main properties: the superior ability to form a stable colloidal suspension in water-based solutions, as highlighted by the measurements of DLS (Table 1), the reactive surface characterized by the presence of a larger amount of –OH groups, useful for an efficient functionalization, as demonstrated by the studies of –OH quantification (Table 3). The ability of the SH0 sample to act as an optical transducer of biomolecular interactions was investigated by studying selective biotin-streptavidin recognition.

Biotin, a member of the water-soluble B-complex group of vitamins, is a molecule involved in a wide range of metabolic processes in humans and other organisms. Due to the strong affinity of biotin to streptavidin, it is commonly used as a model of interaction for the development of diagnostic tools. Once the biotin slides into the tight-fitting pocket of streptavidin, the flexible loop of streptavidin folds over the biotin and acts as a lid for stable binding [23].

**Figure 5:**
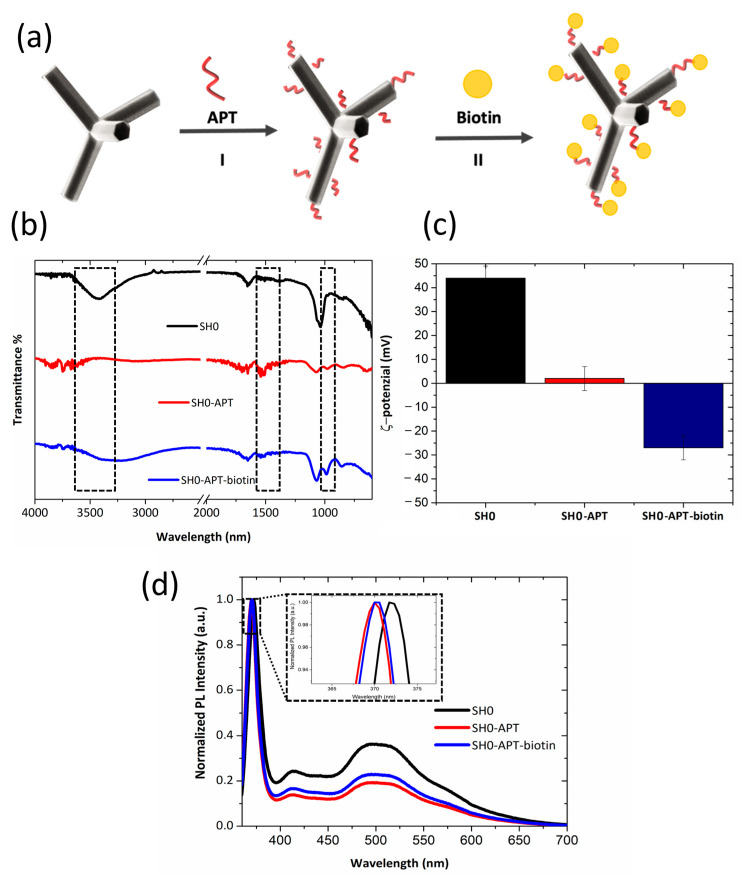
(**a**) Schematic representation of the ZnO-T surface functionalization with APT (**I**) and biotin (**II**). (**b**) FTIR spectrum of ZnO-Ts after each step of functionalization. (**c**) ζ-potential analysis. (**d**) PL spectra of ZnO-Ts before and after the surface functionalization.

Biotin bioprobes were immobilized on the surface of the SH0 ZnO-Ts following the functionalization procedure schematized in Figure 5a. Firstly, the bare ZnO-Ts were silanized by the chemical reaction between the triethoxy groups of APT and the −OH groups on the ZnO-TS surface, generating self-assembled monolayers covalently bonded to the surface via Zn–O–Si bonds able to passivate the surface. This chemical strategy improves the surface stability and introduces coupling points (–NH_2_ groups) for the immobilization of the bioprobe [11,37]. The surface was then biotinylated by the reaction between the N-Hydroxysuccinimide (NHS) esters of biotin (NHS-biotin) molecules and the amine groups of the ZnO-Ts via carbodiimide chemistry at slightly alkaline conditions (pH 8), yielding stable amide bonds.

The chemical modification of the ZnO-T surface was analyzed by ATR-FTIR spectroscopy (Figure 5). As already observed by the graph reported in Figure 1, the spectrum of bare ZnO-Ts is characterized by a broad band at 3500 cm^−1^ due to the presence of –OH hydroxyl groups on the sample surface [25]. After the silanization process, the ZnO-Ts–APT displayed a decrease in the signals related to –OH groups involved in the covalent bond with the silane compound. The characteristic bands of APT, corresponding to the bending mode of free NH_2_ at 1520–1330 cm^−1^ and the rocking CH_x_ vibration of the Si–OCH_x_ bond at 870 cm^−1^, are well evident in the spectrum of the silanized sample. After the biotin immobilization, no significant changes in the FTIR spectrum were observed. The surface charge of the bare ZnO-Ts passed from −45 ± 10 mV to 5 ± 2 mV after the silanization process and to −27 ± 18 mV after biotinylation, confirming the success of the ZnO-T surface functionalization. The effectiveness of the surface functionalization was also demonstrated by photoluminescence (PL) measurements performed by exposing the sample to UV laser light (λ = 325 nm) and analyzing its emission spectrum after each functionalization step. In these investigations, the sample was deposited on a silicon piece. PL measurements were preferred to standard fluorescence investigations performed in solution because they allow exploring a wider spectral range with higher resolution due to the use of high-performing source and detector devices. Figure 5d shows the PL spectra of ZnO-Ts before and after each functionalization step. Compared to the fluorescence spectrum (λ_exc_ = 370 nm) of bare SH0 reported in Figure 2b, its corresponding PL spectrum excited at 325 nm allows observing an intense peak at 372 nm, which can be assigned to the radiative recombination of the electron-hole pairs due to the transition from the conduction band to the valence band (excitonic emission). The visible broad band that peaked at 500 nm (green emission), related to the recombination of electrons with photo-generated holes occupying defect sites, is also well evident [38].

After the silanization process, a slight shift of the peak at 372 nm towards shorter wavelengths (Δλ = −2 nm) was observed; the shift can be attributed to the coordination between the APT molecules and ZnO, which affects the band gap of the material. On the other hand, the decrease in the green emission intensity (about 40%) was due to the binding of the APT molecules with the ZnO-Ts through the –OH groups, reducing the availability of surface holes (i.e., the recombination centers) and, consequently, the intensity of green emission. This result agrees with the FTIR measurements highlighting the decrease in –OH groups after the silanization.

After the functionalization with biotin, only a weak increase in the green emission intensity (about 15%) was monitored.

The biotinylated ZnO-Ts can interact with streptavidin, a homo-tetrameric (66 kDa) protein from the bacterium *Streptomyces avidinii* with an extraordinary affinity to biotin with a dissociation constant (K_d_) in the order of ≈10^−14^ mol L^−1^. The binding of biotin to streptavidin is one of the strongest non-covalent interactions known in nature, forming the basis for many diagnostic assays that require the formation of a specific linkage between biological macromolecules [23].

**Figure 6:**
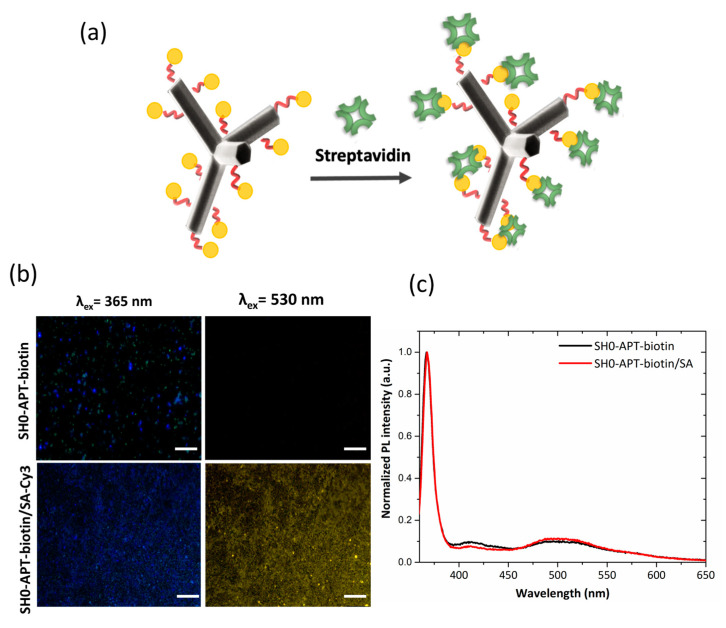
(**a**) Schematic representation of the streptavidin detection by biotinylated-ZnO-Ts. (**b**) Fluorescence microscope images of functionalized SH0 before and after the interaction with Cy3-labeled streptavidin. Scale bar 75 μm. (**c**) Normalized PL intensity spectra of functionalized ZnO-Ts before and after the exposure to label-free streptavidin at the excitation wavelength of 325 nm.

To verify the interaction between biotinylated ZnO-Ts and streptavidin, functionalized ZnO-Ts were preliminary incubated with 0.4 mM of Cy3-labelled streptavidin solution (PBS, pH 8) for 1 h under stirring (Figure 6a). After the incubation, biotinylated ZnO-Ts were washed, deposited on a silicon piece, left to dry, and analyzed by fluorescence microscopy. Figure 6b reports the fluorescence images of biotinylated ZnO-Ts before and after the incubation with fluorescent streptavidin under two different excitation wavelengths, 365 and 530 nm, respectively. The typical yellow emission of Cy3, well evident in the case of biotinylated ZnO-Ts incubated with Cy3-labeled streptavidin under an excitation of 530 nm, confirms the biomolecular interaction.

An analogue investigation was also performed using a label-free streptavidin; in this case, the biomolecular recognition was monitored by PL analysis. The analysis did not reveal any variation in the PL spectrum.

We can conclude that the analysis of the photoluminescence emission can be a valid strategy for studying the surface functionalization of ZnO-Ts; indeed, the results are consistent with the preliminary FTIR characterization. On the contrary, the technique did not provide information on the interaction between the biotinylated ZnO-Ts and streptavidin. The weak sensing capability could be attributed to the low specific surface area (up to 10 m^2^ g^−1^) that characterizes this nanostructured material compared to other forms of nanostructured ZnO such as ZnO-NWs [39] and porous ZnO [40].

## 3. Materials and Methods

### 3.1. Materials and Reagents

Phosphate-buffered saline tablets (PBS CAS No.: P4417-50), Tween 20 (CAS No.: 9005-64-5), 3-aminopropyltriethoxysilane (APT CAS No.: 919-30-2), DMT-dT Phosphoramidite (CAS No.: 98796-51-1), tetrazole (CAS No.: 919-30-2288-94-8), deblocking solution of trichloroacetic acid in dichloromethane 3% *w/w* (CAS No.: 8-570-14); tetrahydrofuran anhydrous (THF CAS No.: 109-99-9), acetonitrile (CAS No.: 75-05-8), hydrochloric acid (HCl CAS No.: 7647-01-0), and sodium hydroxide solution (NaOH CAS No.: 1310-73-2) were all purchased from Sigma-Aldrich. Dimethyl sulfoxide (DMSO CAS No.: 67-68-5), sulfo-N-hydroxysuccinimide biotin (biotin-NHS CAS No.: 119616-38-5) water-soluble, streptavidin (SA CAS No.: 9013-20-1) from *Streptomyces avidinii*, and streptavidin−Cy3 (Cy3-SA CAS No.: S6402) from *Streptomyces avidinii* were purchased by Merck KGaA (DE). Hoechst 3342 Trihydrochloride Trihydrate-10 mg/mL solution (Hoechst CAS No.: 23491-45-4) in water was purchased from Invitrogen by Thermo Scientific. Absolute ethanol (EtOH CAS No.: A3678) was purchased from PanReac Applichem ITW Reagents. Ultra-pure water (18 Ω·cm) purified from a Milli-Q purification system (Millipore, Bedford, MA, USA) was used to prepare all the aqueous solutions.

### 3.2. ZnO Tetrapods (ZnO-Ts) Synthesis

ZnO-Ts were synthesized in a vertical reactor with heating similar to a combustion method described earlier [41]. Briefly, micron-sized Zn particles, entrained in the air, were supplied to the reactor and combusted to form a nanopowder of ZnO. Differently from the previous method, heating from the combustion of methane gas was used instead of electrical heating, and wet air was additionally supplied to the reactor to control the reaction rate. The nanopowder containing ZnO-Ts and some other forms on nanowires and nanoparticles were collected downstream of the reactor on the cellulose filter and further used in the analysis. The tetrapodal shape of the ZnO nanomaterials obtained was confirmed by in-depth TEM analysis in earlier work [41] using the same synthesis conditions. The combustion synthesis method was used because it delivers the synthesis of chemically pure ZnO-Ts in high yield, which is especially attractive for further practical application [10].

After synthesis, the as-obtained ZnO-Ts mixture was separated into 3 fractions with the help of a centrifuge (Table 4). The initial ZnO-T mixture was marked as SH0; it was suspended in isopropanol (IPA CAS No.: 67-63-0) at a concentration of 1 mg/mL, sonicated in the bath for 1 h, and further separated in the centrifuge at a rotation speed of 1000 rpm. The sediments were collected and marked as SH1. The supernatant was further centrifuged at a rotation speed of 3000 rpm; the sediments were then collected and marked as SH2. All fractions were characterized without further processing.

### 3.3. ZnO-Ts Characterization

*Dynamic light scattering (DLS).* Each sample of ZnO-Ts (SH0, SH1, and SH2) was prepared for DLS characterization as described in the following. A stock suspension of ZnO-Ts with a concentration of 0.2 mg mL^−1^ was obtained by dispersing the powder in PBS 1× + 0.1% Tween 20 (PBS-T). Then, a sample with a concentration of 0.05 mg mL^−1^ was prepared by dispersing an aliquot of the stock suspension in ultra-pure water (pH = 7). The sample was centrifuged at 3500 rpm for 3 min and resuspended in ultra-pure water to completely remove the PBS-T. Before the DLS analysis, the sample was sonicated for 5 min. Hydrodynamic diameter and surface ζ-potential of ZnO-Ts were measured using a Zetasizer Nano-ZS instrument (Malvern Instrument Ltd., UK) equipped with a He-Ne laser (633 nm, scattering angle of 90°, 25 °C). Size distribution and surface zeta-potential values were obtained by averaging three measurements.

*UV-Vis spectroscopy.* Absorption spectra of ZnO-Ts were acquired using a Jasco V-730 UV-Vis double beam spectrophotometer (Jasco Inc., Easton, PA, USA) in the wavelength range of 280–800 nm. The samples were analyzed at the concentration of 0.2 mg mL^−1^ in PBS-T using a quartz cell with a path length of 10 mm and a total volume capacity of 0.5 mL.

*Fluorescence spectroscopy.* Fluorescence emission spectra of ZnO-Ts were acquired using a PerkinElmer LS 55 Luminescence spectrometer (PerkinElmer Inc., Waltham, MA, USA) in the wavelength range of 380–700 nm, setting the excitation wavelength at 370 nm, and the excitation and emission bandwidth at 10 nm and 5 nm, respectively. The samples were suspended in PBS-T, and a quartz cell with a path length of 10 mm and a total volume capacity of 0.5 mL was used.

*Fluorescence microscopy.* Fluorescence images of ZnO-Ts were acquired using a Leica AF6000LX-DM6M-Z microscope (Leica Microsystems, Mannheim, Germany), controlled by LAS-X (Leica Application Suite; rel. 3.0.13) software and equipped with a DFC7000T Leica Camera. Fluorescence images were obtained using a 50× objective and an I3 cube constituted by a 365 nm band-pass excitation filter. ZnO-Ts were dispersed in ultra-pure water (250 mg mL^−1^), and 20 μL of the samples were left to dry on silicon pieces to acquire the images.

*Brunauer-Emmett-Teller (BET) analysis*. The textural parameters of the samples were determined by nitrogen adsorption–desorption isotherms at −200 °C (77 K) using a Quantachrome Autosorb-iQ-KR/MP automated gas sorption analyzer. Before the analysis, the powder samples were outgassed under a vacuum at 200 °C for 3 h. The specific surface area was calculated using the BET (Brunauer–Emmett–Teller) equation.

*Transmission Electron Microscopy.* The morphology of the samples SH0, SH1, and SH2 was investigated using a transmission electron microscope (TEM, Jeol JEM-1400, Jeol Ltd., Akishima, Japan). To this aim, the samples were dispersed in ultra-pure water at a concentration of 0.2 mg mL^−1^ and dropped on a carbon-coated copper TEM grid before air-drying overnight at room temperature.

*ATR-Fourier Transform Infrared Spectroscopy.* The surface chemical composition of ZnO-Ts was investigated by attenuated total reflectance Fourier transform infrared spectroscopy (ATR–FTIR). The ATR– FTIR spectra of the samples were obtained using a Nicolet iS50 (Thermo Scientific) FTIR spectrometer equipped with a Germanium (Ge) crystal element. The ATR–FTIR spectra were recorded in the wavenumber region 4000–525 cm^−1^ with a resolution of 4 cm^−1^. The measurements were carried out on dried ZnO-Ts deposited on the Ge crystal.

*Photoluminescence.* The photoluminescence analysis of ZnO-Ts was performed by depositing 20 μL of the samples dispersed in ultra-pure water (0.2 mg mL^−1^) on silicon pieces; the samples were left to dry at RT before the investigations. The photoluminescence (PL) spectra of ZnO-Ts were excited by a continuous wave He-Cd laser at 325 nm (KIMMON Laser System). PL was collected at normal incidence to the surface of samples through a fiber, dispersed in a spectrometer (Princeton Instruments, SpectraPro 300i), and detected using a Peltier-cooled charge-coupled device (CCD) camera (PIXIS 100F). A long pass filter with a nominal cut-on wavelength of 350 nm was used to remove the laser line at the monochromator inlet.

### 3.4. Quantum Yield (QY) Calculation

Quantum yields (QYs) of the samples SH0, SH1, and SH2 dispersed in PBS-T were calculated by measuring their absorbance and the integrated fluorescence intensity at different concentrations (0.05, 0.1, 0.15, and 0.2 mg mL^−1^). Absorption spectra were obtained using a Jasco V-730 UV-Vis double beam spectrophotometer (Jasco Inc., Easton, PA, USA) in the wavelength range of 280–800 nm. Emission spectra were acquired using a PerkinElmer LS 55 Luminescence spectrometer (PerkinElmer Inc., Waltham, MA, USA) in the wavelength range of 410–600 nm, setting the excitation wavelength at 370 nm. The QYs of ZnO-Ts were estimated relative to Hoechst 33342 (Hoechst) used as a standard dye. To this aim, Hoechst was dispersed in DMF at the concentrations of 0.0075, 0.015, 0.025, and 0.050 mg mL^−1^ and analyzed.

### 3.5. Quantification of -OH Groups

ZnO-T samples SH0, SH1, and SH2 (20 mg) reacted with tetrazole activated (18 mg) 5′-(dimethoxytrityl)-thymidine-phosphoramidite dissolved in dry THF (30 mg/mL) for 1 h at room temperature under mild stirring [34].

Then, the samples were centrifuged for 3 min at 5000 rpm and washed 10 times with acetonitrile to remove adsorbed reagents. The removal of the 5′-dimethoxytrityl protecting group from the supports bound 5′-terminal nucleotide was performed by using the deblocking solution of dichloroacetic acid in dichloromethane (3% *w/w*). The ZnO-Ts were centrifuged at 5000 rpm, and the amount of DMT in the supernatant was measured by a UV-Vis spectrometer (V-73, Jasco Europe, Italy) at 503 nm (ε = 71,700 M^−1^ cm^−1^). Functionalization of the samples was performed in triplicate.

### 3.6. ZnO-Ts Stability at Different pH Conditions

To investigate the stability of ZnO-Ts on exposure to acidic and alkaline conditions, ZnO-Ts were dispersed at a concentration of 0.2 mg mL^−1^ in PBS-T at different pHs (3, 4, 5, 6, 7, and 8) for 0, 2, and 24 h. After the exposure, the samples were investigated by absorbance, fluorescence, and ATR-FTIR analyses.

### 3.7. ZnO-Ts Functionalization

The studies of functionalization were performed on the SH0 sample. ZnO-Ts (1 mg) of SH0 were amino-modified using APT 5% (*v/v*) in absolute EtOH (1 mL final volume) for 1 h at room temperature (RT) in mild stirring conditions [42]. The sample was centrifuged at 3500 rpm, and the supernatant was replaced twice with EtOH and once with PBS (pH = 8) to remove unreacted APT. Amino-modified ZnO-Ts (1 mg) were resuspended in biotin-NHS solution (1 mM in PBS, pH 8) and left to react for 1 h [23].

ZnO-Ts were characterized after each functionalization step by DLS, photoluminescence, and ATR-FTIR analyses.

### 3.8. Sensing Experiment

Two aliquots of biotinylated ZnO-Ts were incubated with 0.4 mM of SA and Cy3-SA and left to interact for 1 h with an agitation of 800 rpm. After the interaction, the samples were washed and resuspended in PBS (pH = 8).

The interaction between the biotinylated ZnO-Ts and the streptavidin was monitored by fluorescence microscopy and photoluminescence spectroscopy.

## 4. Conclusions

The growing advancement of the biosensor field provides a powerful driving force for the constant research and fabrication of advanced nanostructured materials with enhanced properties for developing a new generation of diagnostic devices. Zinc oxide (ZnO) is one of the most interesting metal oxide materials used in biosensing due to its unique and versatile physicochemical properties. It has been observed that the properties of ZnO can be improved through the nanoscale-up architectural process, making ZnO-based structures suitable for biosensing applications and opening the way for the development of multiparametric, label-free transducers. In this work, we discussed the potentiality and performance of various novel ZnO-tetrapod nanostructures for label-free optical biosensing applications. The physical and chemical properties of three different ZnO-T samples (SH0, SH1, and SH2), synthesized by the combustion process and separated by a centrifugation method in different size fractions, were evaluated by DLS, TEM, BET analyses, FTIR, UV-vis spectroscopy, spectrofluorimetry, steady-state PL, and fluorescent microscopy. Then, we explored the chemical reactivity of the samples by surface coupling with monomeric oligonucleotide bases via the phosphoramidite method and quantified the available functional hydroxyl groups (–OH) on the transducer surface necessary for the subsequent functionalization steps. The best ZnO-T sample in terms of colloidal stability, PL quantum yield, and chemical reactivity was chemically modified and bioconjugated with biotin using chemical protocols able to preserve the physicochemical properties of the matrix based on silanization and carbodiimide chemistry. FTIR, zeta potential and PL analyses confirmed the successful obtainment of the biotinylated ZnO-T-based biosensor. The sensing properties of the obtained device were investigated by optical methods based on the steady-state PL and fluorescence microscopy, confirming the biotin-streptavidin interaction. Although further modifications in the synthetic process will have to be carried out to implement the chemical-physical properties of these emerging structures for label-free biosensing applications, the detailed analysis of ZnO nanostructures conducted in this study will contribute to future biosensing applications of these appealing structures.

## Figures and Tables

**Table 1:** Hydrodynamic diameter (size), polydispersity index (PdI), and surface charge (zeta potential) of ZnO-T samples obtained by DLS measurements. The analyses were performed in ultra-pure water (pH 7).

Sample	Size (nm)	PdI	Zeta Pot (mV)
SH0	330 ± 120	0.3	−47 ± 5
SH1	470 ± 100	0.5	−40 ± 5
SH2	600 ± 250	0.4	−43 ± 4

**Table 2:** Relative quantum yield values calculated from Equation (1).

Sample	QY%
SH0	4.7 ± 0.6
SH1	14 ± 1
SH2	6 ± 1

**Table 3:** Quantification of reactive hydroxyl (–OH) groups.

Sample	–OH (µmol mg^−1^)
SH0	3.9 ± 0.2
SH1	3.5 ± 0.6
SH2	1.8 ± 0.5

**Table 4:** Fabrication procedures of the ZnO-T samples.

ZnO-Ts Sample	Fabrication Procedure
SH0	as-obtained ZnO-Ts
SH1	SH0 dispersed in IPA, sonicated for 1 h, and centrifuged at 1000 rpm, with the sediment collected and marked as SH1
SH2	The supernatant of SH1 centrifuged at 3000 rpm, with the sediment collected and marked as SH2

## Data Availability

Data is contained within the article and supplementary material.

## Supplementary Materials

The following supporting information can be downloaded at: https://www.mdpi.com/article/10.3390/ijms24054449/s1.
