# Supplementary material for: ZnO Tetrapods for Label-Free Optical Biosensing: Physicochemical Characterization and Functionalization Strategies
Source: Int J Mol Sci. 2023 Feb 23;24(5):4449. doi: 10.3390/ijms24054449 (PMC10002590; doi:10.3390/ijms24054449)
Supplement: Supplementary file 1 [file ijms-24-04449-s001.zip › ijms-2194422-supplementary.pdf]

ZnO Tetrapods for Label-Free Optical Biosensing: Physicochemical Characterization and Functionalization Strategies

Monica Terracciano [1,†], Simas Račkauskas [2,†], Andrea Patrizia Falanga [1], Sara Martino [3,4], Giovanna Chianese [3], Francesca Greco [1], Gennaro Piccialli [1], Guido Viscardi [5], Luca De Stefano [3], Giorgia Oliviero [6], Nicola Borbone [1,*] and Ilaria Rea [3]

[1] Department of Pharmacy, University of Naples Federico II, Via Domenico Montesano 49, 80131 Naples, Italy
[2] Institute of Materials Science, Kaunas University of Technology, 51423 Kaunas, Lithuania
[3] Unit of Naples, National Research Council, Institute of Applied Sciences and Intelligent Systems, Via Pietro Castellino 111, 80131 Naples, Italy
[4] Department of Precision Medicine, University of Campania "Luigi Vanvitelli", 80138 Naples, Italy
[5] Department of Chemistry, NIS Interdepartmental Centre, University of Turin, Via Pietro Giuria 7, 10125 Turin, Italy
[6] Department of Molecular Medicine and Medical Biotechnologies, University of Naples Federico II, Via S. Pansini 5, 80131 Naples, Italy
* Correspondence: nicola.borbone@unina.it; Tel.: +39-081678521
† These authors contributed equally to this work.

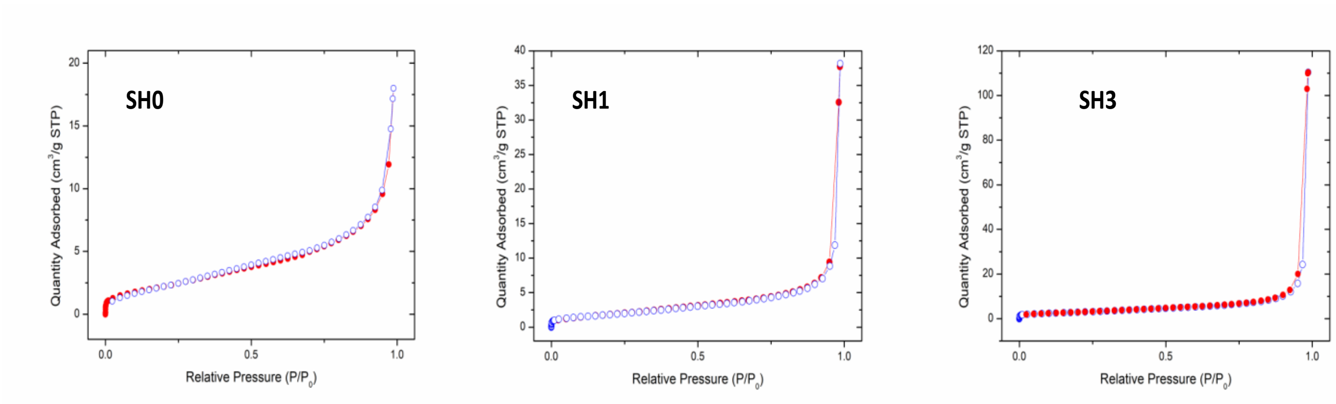

Figure S1. BET analysis.

After immersion of ZnO in deionized water, the hydrolysis of the particles surface occurs as a result of the adsorption of water molecules with the simultaneous formation of $Zn(OH)_{2\,(s)}$ layer. The zinc hydroxide is soluble in water becoming more soluble as pH is lowered or increased. The predominant reactions are the dissolution of $ZnO_{(s)}$ to $Zn^{2+}_{(aq)}$ and $O^{2-}_{(aq)}$ and surface hydroxylation to $Zn(OH)^{+}_{(s)}$ following Equations (S1)–(S2).

$$ZnO_{(s)} \leftrightarrow Zn^{2+}_{(aq)} + O^{2-}_{(aq)} \text{ metal oxide dissolution (S1)}$$

$$ZnO_{(s)} + H^{+}_{(aq)} \leftrightarrow Zn(OH)^{+}_{(s)} \text{ metal oxide hydroxylation (S2)}$$

When the pH is lowered from pH 7.7 to 4.3, the following reactions taken place (Equations (S3)–(S7)).

$$Zn(OH)^{+}_{(aq)} \leftrightarrow Zn^{2+}_{(aq)} + OH^{-}_{(aq)} \text{ (S3)}$$

$$Zn(OH)_{2(s)} \leftrightarrow Zn(OH)^{+}_{(aq)} + OH^{-}_{(aq)} \quad (S4)$$
$$Zn(OH)_{2(s)} \leftrightarrow Zn(OH)_{2(aq)} \quad (S5)$$
$$Zn(OH)_{2(s)} \leftrightarrow Zn^{2+}_{(aq)} + 2OH^{-} \quad (S6)$$
$$O^{2-}_{(aq)} + 2H^{+} \leftrightarrow H_2O \ (S7)$$

In highly acidic suspensions, the H^+ ions from HCl will react with the ZnO surface causing the complete dissolution of ZnO and forming $Zn^{2+}_{(aq)}$and H_2O.

In the alkaline region 7.7 < pH < 11.6, the dissolution of ZnO is related to its hydroxide which produces soluble species in the form of hydroxyl complexes such $Zn(OH)_{2(aq)}$, $Zn(OH)^{-}_{3(aq)}$, and $Zn(OH)^{2-}_{4(aq)}$, according to the following reactions (Equations (S8)–(S12)).

$$Zn^{2}_{(aq)} + OH^{-} \leftrightarrow Zn(OH)^{+}_{(aq)} \quad (S8)$$
$$Zn(OH)^{+}_{(aq)} + OH^{-} \leftrightarrow Zn(OH)_{2(s)} \ (S9)$$
$$Zn(OH)_{2(s)} \leftrightarrow Zn(OH)_{2(aq)} \quad (S10)$$
$$Zn(OH)_{2(s)} + OH^{-} \leftrightarrow Zn(OH)^{-}_{3(aq)} \quad (S11)$$
$$Zn(OH)_{2(s)} + 2OH^{-} \leftrightarrow Zn(OH)^{2-}_{4(aq)} \ (S12)$$
